# Landscape Ecology and Epidemiology of Malaria Associated with Rubber Plantations in Thailand: Integrated Approaches to Malaria Ecotoping

**DOI:** 10.1155/2015/909106

**Published:** 2015-03-09

**Authors:** Wuthichai Kaewwaen, Adisak Bhumiratana

**Affiliations:** ^1^Department of Geoinformatics, Faculty of Geoinformatics, Burapha University, Chonburi 20131, Thailand; ^2^Department of Parasitology and Entomology, Faculty of Public Health, Mahidol University, 420/1 Rajvithi Road, Rajthewee, Bangkok 10400, Thailand; ^3^Center for Ecohealth Education and Research (CEER), Faculty of Public Health, Thammasat University, Rangsit Center, Pathum Thani 12121, Thailand

## Abstract

The agricultural land use changes that are human-induced changes in agroforestry ecosystems and in physical environmental conditions contribute substantially to the potential risks for malaria transmission in receptive areas. Due to the pattern and extent of land use change, the risks or negatively ecosystemic outcomes are the results of the dynamics of malaria transmission, the susceptibility of human populations, and the geographical distribution of malaria vectors. This review focused basically on what are the potential effects of agricultural land use change as a result of the expansion of rubber plantations in Thailand and how significant the ecotopes of malaria-associated rubber plantations (MRP) are. More profoundly, this review synthesized the novel concepts and perspectives on applied landscape ecology and epidemiology of malaria, as well as approaches to determine the degree to which an MRP ecotope as fundamental landscape scale can establish malaria infection pocket(s). Malaria ecotoping encompasses the integrated approaches and tools applied to or used in modeling malaria transmission. The scalability of MRP ecotope depends upon its unique landscape structure as it is geographically associated with the infestation or reinfestation of *Anopheles* vectors, along with the attributes that are epidemiologically linked with the infections. The MRP ecotope can be depicted as the hotspot such that malaria transmission is modeled upon the MRP factors underlying human settlements and movement activities, health behaviors, land use/land cover change, malaria vector population dynamics, and agrienvironmental and climatic conditions. The systemic and uniform approaches to malaria ecotoping underpin the stratification of the potential risks for malaria transmission by making use of remotely sensed satellite imagery or landscape aerial photography using unmanned aerial vehicle (UAV), global positioning systems (GPS), and geographical information systems (GIS).

## 1. Overview of Malaria Epidemiology Landscape Changes and Risks

At present, global environmental changes have been linked to the geographical distribution and dynamics of malaria. As with the simulations, the depictions of anthropogenic global and regional climate changes on health impact of malaria can alter malaria epidemiology landscape in receptive areas of Southeast Asia, South America, and East Africa [1–5]. Such climate changes driven by greenhouse gas and land use change have been projected to significantly affect the spread of malaria in tropical Africa before 2050 [4]. The causal links between environmental change and human health are complex because the cause-effect relationships are often indirect and dynamic over space and time. Malaria is caused by five human malarial parasites including* Plasmodium falciparum*,* P. vivax*,* P. malariae*,* P. ovale*, and* P. knowlesi*. Its naturally complete transmission requires human and vector systems. The malaria ecology is the integrated science of studying the interactions of malaria parasites circulating in their reservoir hosts,* Anopheles* vectors and humans, and the constraints of which the adaptation of malaria parasites is regulated by the activities of humans and* Anopheles* vectors, as well as by the biological and physical environments of* Anopheles* vectors in a niche, a habitat, or an ecosystem. Actually, the dynamics of the malaria ecology is constrained geographically and seasonally by ecological relationships in nature. 

What happened to breakdown the dynamics of the malaria ecology stems from two phenomena, which include anthropogenic climate and land use changes [6–14]. Only the land use change is profoundly addressed here. Apart from that are driven by the natural processes, the land use changes driven by human activities have the potential effects on climate, whether globally, regionally, or locally, and can modify or manipulate the ecology of malaria as well as other vector-borne diseases [8, 15–17]. The main drivers contributing greatly to malaria epidemiology landscape changes include deforestation, dam construction, irrigation, stream diversion, agricultural land use change, and unplanned urbanization [8–14]. Having the potential effects on the health impact of malaria, these human-induced changes can induce direct changes in the geographical distribution of malaria incidence and* Anopheles *vectors [6, 7, 9, 10, 13, 18–21], as well as the vectorial capacity of* Anopheles *vectors [22, 23]. Also, such indirect changes in environmental conditions including shaded environments affect the availability of breeding sites and feeding activities [24, 25]. Taken together, if expected to accompany climate change or other human-induced changes, such anthropogenic land use change contributes substantially to the potential risks for malaria transmission in receptive areas due to the combination of malaria transmission dynamics, the susceptibility of human populations, and the geographical distribution of malaria vectors.

In present review, the authors focused radically on the expansion of rubber plantations as the agricultural land use changes by delineating a unique landscape structure (i.e., the pattern and extent) of the ecotope of malaria-associated rubber plantations (MRP). This agricultural intensification is considered the main driver that has the potential effects on malaria transmission dynamics occurring in forest-related and forest fringe-related malaria in Thailand [26, 27]. The MRP ecotope, on the other hand, can be depicted as the hotspot of modeling malaria transmission dynamics upon the MRP factors underlying human settlements and movement activities (e.g., revisiting rubber plantation polygon(s) and routine rubber plantation practices), health behaviors, land use/land cover change, malaria vector population dynamics, and agrienvironmental and climatic conditions. Of note, this review synthesized the novel concepts and perspectives on applied landscape ecology and epidemiology of malaria, as well as approaches to determine the degree to which the MRP ecotope as fundamental landscape scale can establish malaria infection pocket(s). The challenge is that the advancement of malaria ecotoping in any hotspots pertaining to malaria epidemiology landscape change is to integrate systemic and uniform approaches and tools for modeling malaria transmission by making use of remotely sensed satellite imagery or landscape aerial photography using unmanned aerial vehicle (UAV), global positioning systems (GPS), and geographical information systems (GIS).

## 2. Current Status of Forest-Related and Forest Fringe-Related Malaria Landscape in Thailand

Thailand is located in the Southeast Asia and bordered by Cambodia, Lao People's Democratic Republic, Myanmar, and Malaysia. The country is also constituted of the Greater Mekong Subregion (GMS) that includes Cambodia, Lao PDR, Myanmar, People's Republic of China (Yunnan, PRC), Thailand, and Vietnam. It has a land area of 51,311,501.92 hectares (approximately 513,115 km^2^) (see Table S1 in Supplementary Material available online at http://dx.doi.org/10.1155/2015/909106) and a population of 64,785,909 people as of 2013 [28]. From the 1980s to the 1990s, Thailand had lost forest cover of between 13.0 million hectares and 14.8 million hectares. However, in the early 2010s, the forest land has been increased up to 17.6 million hectares as a result of the continuation of the policy implementation on reforestation, rehabilitation, and restoration across five regions of the country (see Table S1).

The propagation of upland perennial agriculture attributed to human activities has the potential effects on reduction or loss of biodiversity and habitats, surface water hydrology, soil erosion, and carbon sink and flux [11–14]. For instance, the expansion of perennial agriculture (e.g., rubber and other mixed oil palms or orchards) is a trade-off if expected to accompany land reform and human settlement/resettlement through the policy-driven economy and social/human development [27]. This phenomenon is explained by the disturbance and fragmentation of the forest land affected by increasing agricultural land. That is, the affected forest land is influenced by which the perennial agriculture, whether or not it is irrigated, is cultivated around or close to the forests and to which human settlements and activities are related. However, this agricultural land use change is in turn a driving force influencing the functioning ecosystem and services of the forests and the connectivity of forest patches.

**Figure 1:**
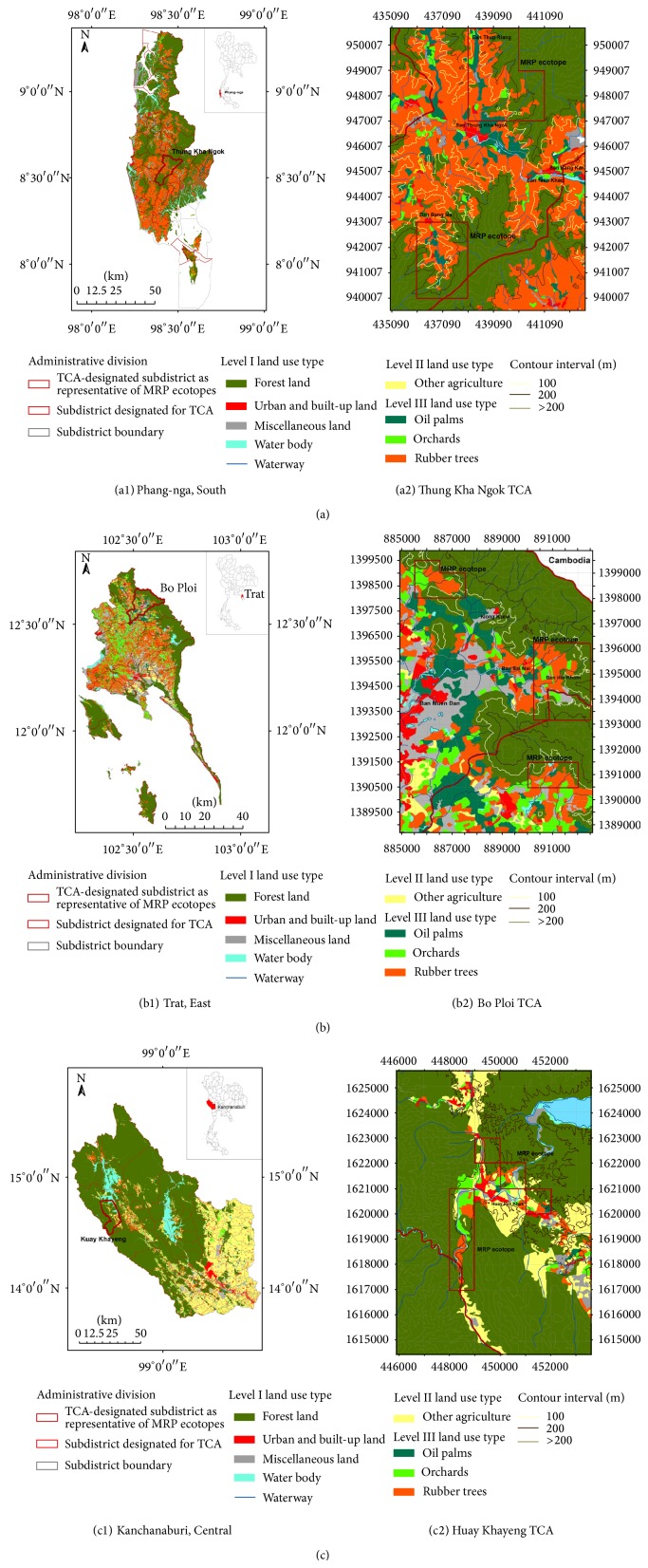
Transmission control areas (TCAs) establishing MRP ecotopes illustrated with contour lines. As the result of the expansion of rubber plantations, three different malaria-endemic provinces of Thailand, Phang-nga (a), Trat (b), and Kanchanaburi (c), are demonstrated by the affected TCAs at the subdistrict level. (a1) TCAs of Phang-nga virtually established MRP ecotopes. (a2) Thung Kha Ngok TCA shows forest and forest fringe landscape shaped by traditional rubber plantation practices on different altitudes, and two diverse MRP ecotopes with different land use types are shown. (b1) Similar to that occurred in Phang-nga, TCAs of Trat established diverse MRP ecotopes. (b2) Bo Ploi TCA influenced by diverse MRP ecotopes is shown. (c1) Kanchanaburi recently established MRP ecotopes with nontraditional rubber plantation practices, confined within some affected TCAs. (c2) Huay Khayeng TCA influenced by diverse MRP ecotopes is also shown for the potential of malaria transmission risks. All the land use maps that were also validated by the ground surveys between 2013 and 2014 were constructed using the ArcGIS ver. 10.1 software applications. The spatial data were initially obtained from the geospatial imagery by the Landsat 5 satellite.

Here, the propagation of rubber plantations is used as an example of land use/land cover change that has the potential effects on the risks for malaria transmission in Thailand or elsewhere in the GMS countries including Myanmar and Malaysia [26, 27, 29, 30]. The land area covered with as many as rubber plantation polygons [27] is intensively exploited by land management strategy to propagate rubber plantations by private-owned smallholdings or estates. This topographically shaped landscape scale can be specifically defined by a unique landscape structure. In Thailand, this is a topic of interest because the people not only exploit suitable and sustained productions of the natural rubber and wood but also pose the risks for malaria, especially in transmission control areas (TCAs) of the South and East of Thailand as shown in Figures 1(a) and 1(b). This phenomenon can lead not only to the changes of malaria landscape ecology and epidemiology, but also to the consequences of the implementation of currently available malaria control strategies, as well as surveillance systems and tools, at both national and subnational levels within the endemic GMS countries implementing the National Malaria Control Programs (NMCPs) [26, 27, 30, 31].

## 3. MRP Landscape Epidemiology 

### 3.1. Malaria Risks Attributed to the Expansion of Rubber Plantations

Malaria is one of the most important mosquito-borne diseases in Thailand. The disease is caused by two main human malaria parasites,* P. falciparum* and* P. vivax*, and to lesser extent by* P. malariae* and* P. ovale*. Malaria epidemiology normally relates the infections to the causes of malaria in any infected individuals and to the risk factors attributed to malaria among the vulnerable populations involved in agriculture. This malaria epidemiology landscape can be envisaged as the endemic localities—in which local people render the occupational and behavioral exposures susceptible to the infections as those who reach remotely inhabited areas of upland agriculture although infested with malaria vectors. If their settlement is located far from the forest land, the adulthood infection rather than the childhood infection occasionally occurs due to revisiting or performing workmen's forest activities—whether seasonally or periodically—at the forests infested with* Anopheles* vectors [26, 27, 29, 32, 33]. Most malaria-contracted adults acquire the infection through outdoor biting of* Anopheles* vectors during the night time in the absence or lack of preventive measures. If their settlement is located around or close to the forest land, both adulthood and childhood infections occasionally occur through outdoor or indoor biting of* Anopheles* vectors during the night time in the absence or lack of preventive measures [26, 27, 29, 33]. This is a reason why the epidemiologic landscape of forest-related and forest fringe-related malaria relates the schoolchildren and working groups to the occupation involved in agriculture.

The questions are raised about why the risk of MRP occurs only in some valleys or hills and how we could comprehend a topologically detailed and accurate graphic presentation of the expansion of rubber plantation polygons on the valleys and hills, regulating that risk of MRP. To comprehend the pattern and extent of MRP ecotopes that relate malaria transmission risks to the expansion of rubber plantations, we synthesized novel concepts and perspectives on applied landscape ecology and epidemiology of malaria. Understanding a geographically defined malaria ecotope previously described by Sorosjinda-Nunthawarasilp and Bhumiratana [32] is essential to demarcate the scope of any MRP ecotope. The MRP ecotope can be defined as a land area covered with the patches of forests and rubber plantations as it is geographically associated with the infestation or reinfestation of* Anopheles* vectors.

Figure 1 shows the MRP landscape shaped by the expansion of rubber plantations. The MRP ecotope has a unique landscape structure topologically shaped not only by the diverse land use types (or level I to III categories of land use) including rubber trees, whether monocultured or mixed with other perennial trees, waterway, water body, and forests, but also by the natural evaluation and inclination. In Panels (a) to (c), the topographic land use maps show the current status of the expansion of rubber plantations in three different malaria-endemic provinces including Phang-nga (South), Trat (East), and Kanchanaburi (Central). Phang-nga (Panel (a1)) and Trat (Panel (b1)) have many land areas of traditional rubber plantation practices (RPPs), whereas Kanchanaburi (Panel (c1)) exhibits growing trend of increased land areas of nontraditional RPPs. Details of MRP ecotopes with traditional and nontraditional RPPs are described later. Among these malaria-endemic provinces, Phang-nga is a good example of the expansion of rubber plantations into the forest lands such as disturbed forests. A contour map of malaria ecotope or MRP ecotope illustrated with contour lines shows valleys and hills in which malaria transmission is likely to be dynamic as a result of the expansion of rubber plantations on the different elevations within the Thung Kha Ngok TCA of Phang-nga (Panel (a2)).

Since then large-scale development of rubber-planted land area has the effects on shifted land use and land cover, the expansion of rubber plantations has changed, instantly rather than gradually, with the change of the infestation of* Anopheles* vectors. However, the persistence of rubber plantations with dense canopy can gradually recreate shaded environments under suitable agrienvironmental climatic conditions. If connected to the forest land infested with* Anopheles *vectors, the extent of rubber plantation polygons can bring about malaria landscape change. That is, the extended forest cover manages to promote the adaptation and survival of local potent* Anopheles* vectors and human-vector interactions, but not always to facilitate the dispersal of the populations of* Anopheles* vectors although they interact with each other in the habitats with breeding and foraging [32]. However, not all the patches covered with rubber plantation polygons are infested with* Anopheles* vectors. Even if a patch of rubber plantation polygon(s) is infested or reinfested with some* Anopheles* vectors, a probable human-vector contact site is not definitely understood unless malaria or MRP ecotope is well defined. Therefore, we need to understand what are the linkages to which the vulnerable persons including rubber farmers, rubber plantation workers, and accompanied persons acquire the infections over space and time despite the fact that the coverage of malaria control strategies used in the NMCP is household-level implemented.

### 3.2. Malaria Risks Attributed to Rubber Plantation Practices

**Figure 2:**
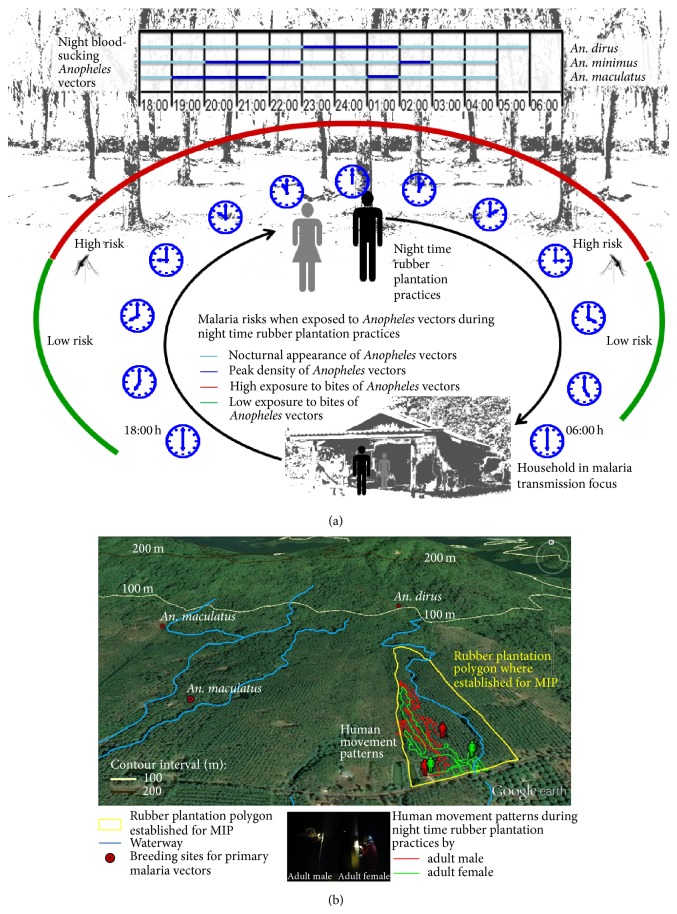
Malaria risks. (a) Malaria risks attributed to rubber plantation practices (RPPs) in the South and East of Thailand. Rubber farmers or rubber plantation workers residing in households or at the smallholdings in the MRP ecotope do not always sleep under nets during the nighttime RPPs. Two hypothetical malaria risks can be depicted for the adulthood infections. The low risk (green) occurs during which the RPPs are performed before 21:00 h or after 03:00 h. The high risk (red) occurs during which the RPPs are continuously performed between 21:00 and 03:00 h. The difference in malaria risks depends on probable human-vector contact through multiple bites of primary* Anopheles* vectors (e.g.,* An. dirus*,* An. minimus*, and* An. maculatus*) at multiple locations. (b) The rubber plantation polygon where established for MIP (also see Figure 5) showing human movement patterns during nighttime RPPs by both adult male (with past history of* P. malariae* infection) (also see Table 2) and female rubber plantation workers. Human movement patterns were recorded using the video recorder compatible with the GPS tracking device, during the ground survey in December 2014. After leaving the house, both female and male workers show movement activities asynchronous to each other.

Figure 2 shows malaria risks are associated with routine RPPs during the night time as often this phenomenon is seen in the MRP ecotope with traditional RPPs in the South and East of Thailand. During seasonal harvestation of the natural rubber, rubber farmers or rubber plantation workers, whether or not they reside in an MRP ecotope, do not always sleep under nets as they have person-time sleeping hours less than that of general people who normally reside in the densely populated areas of the village [27]. However, both low and high malaria risks for the adulthood infection stem from the strong likelihood that probable human-vector contact occurs at multiple locations and time periods during which* Anopheles *vectors seek any blood meals. For example, high malaria risk occurs when any susceptible persons prefer the night time routine RPPs, for example, rubber tapping, rubber coagulation, or rubber sheet processing, at their rubber plantation farm infested with* Anopheles* vectors. During the practices, they do not perceive or beware of the malaria risk by the adoption of personal protection behaviors or boarder preventive measures. That is, if they practice longer time at night, there is the strong likelihood of malaria risk.

Rubber farmers or rubber plantation workers involved in most traditional RPPs normally schedule routine RPPs (e.g., rubber tapping and rubber sheet processing), starting from early wet season and extending to cool season (Figure 2(a)). In a given rubber plantation farm (or a rubber plantation polygon) that produces the natural rubber sheets, skillful rubber plantation workers (2 persons) can tap up to 400 to 500 rubber trees per night and, continually, harvest the rubber milk to process the rubber sheets. Tapping a rubber tree varies from one to two minutes before getting started a new one. If they start to tap the rubber trees around 21:00 h, it is possible that they will acquire frequent exposure to bites of* Anopheles *vectors around 03:00 h or to be exposed continuously to multiple bites from 21:00 to 06:00 h. This is why the probable human-vector contact site is not always close to the house during which seasonal harvestation is operated. The occurrence of probable human-vector contact is likely to be dynamic if there is the availability of* Anopheles* breeding sites and feeding activities on different altitudes and inclinations. The malaria risk is more likely to be dynamic if there is difference in human movement patterns during nighttime rubber plantation practices (Figure 2(b)), which include nighttime rubber tapping (hours) and rubber tapping patterns (days) such as alternate-day tapping and 2-3 days after a day of recovery. If expected to accompany risk behaviors, these human movement activities can pose the risk for malaria infections, depending on how any vulnerable persons acquire the multiple bites of* Anopheles *vectors at multiple locations.

In general, the seasonal malaria transmission relating to seasonal harvestation occurs in most MRP ecotopes of the Southern and Eastern Thailand. The seasonal harvestation normally takes about 10 months of harvestation (starting from May to February of the next year), and then a 2-month of recovery persists between March and April. In the East of Thailand, there is a tendency of schedule change due to local climate change. If the rubber trees shade their leaves as continuously exposed to cool weather, a recovery phase of harvestation will vary from as early as mid January to mid March, and then seasonal harvestation will start as early as in late March. Eventually, the dynamics of malaria transmission occurring in the diverse MRP ecotopes of the Eastern Thailand maybe relate local climate change to the temporal and spatial distributions of vector populations and the seasonal harvestation of the natural rubber. Taken together, the MRP ecotope encompasses the epidemiological complex setting in which the malaria transmission risk is relatively associated with geospatial distributions and behaviors of the* Anopheles *vectors, human settlement, and movement activities such as revisiting rubber plantation polygons infested with* Anopheles *vectors and routine RPPs. That is why we need to comprehend MRP ecotope to better understand the stratification of malaria risks in certain endemic localities or transmission areas for surveillance, prevention, and control.

## 4. MRP Landscape Ecology

### 4.1. MRP Ecotopes with Traditional Rubber Plantation Practices

As illustrated earlier by Figures 1 and 2, land areas covered with traditional rubber plantations in the South and East of Thailand are situated on different altitudes up to 200 meters above sea level (MASL) and slopes up to 35 degrees. The rubber trees manage normally to grow under the climatologically and geologically suitable conditions and to persistently yield the natural rubber after 7 to 20 years of the plantation. On the other hand, the MRP ecotope with traditional RPPs becomes epidemiological complex setting in which the malaria infection pocket (MIP) is likely to be established in space and time through the interconnections that can pose probable human-vector contact.

**Figure 3:**
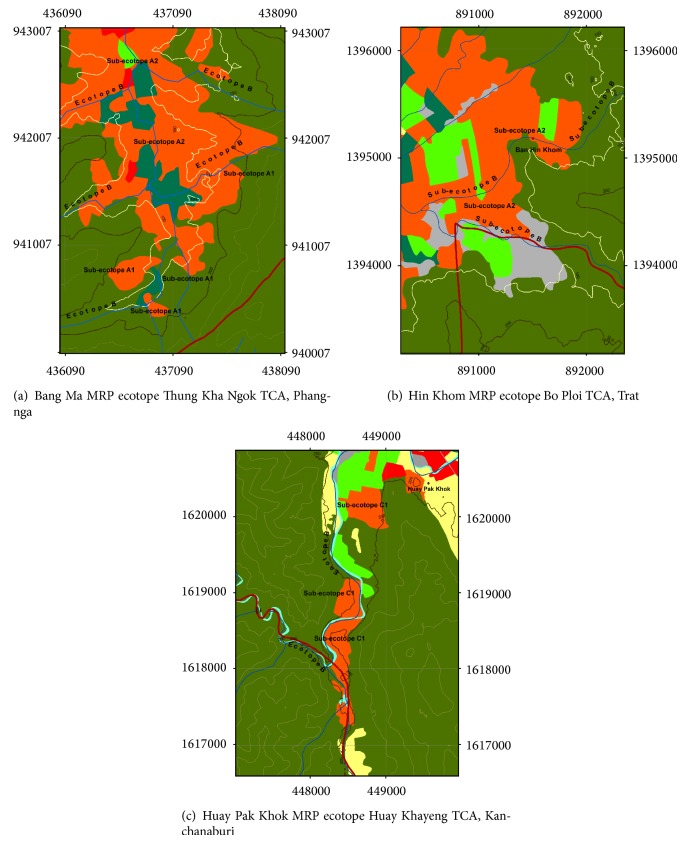
Land use maps showing diverse MRP ecotopes as representative of three malaria-endemic provinces of Thailand. The relative land use types pertaining to the MRP ecotope are shown for (a) the rubber plantation polygons of the subecotopes A1/A2 and the ecotope B in the Bang Ma MRP ecotope; (b) the rubber plantation polygons of the subecotope A2 and the ecotope B in the Hin Khom MRP ecotope; and (c) the rubber plantation polygons of the subecotope C1 and the ecotope B in the Huay Pak Khok MRP ecotope. Landscape structure of the MRP ecotope typically represents the subecotope A1 between the contour lines, 100 and 200 MASL, and the subecotope A2 at the altitude lower than a 100 MASL. All the validated land use maps illustrated with contour lines were constructed as before.

Understanding the topography of diverse MRP ecotopes with traditional RPPs is needed not only to analyze what characteristics of MRP ecotope with traditional RPPs are connected to establish an MIP but also to determine the degree to which local* Anopheles* vectors are adapted to local environment of the MRP ecotope. In this regard, we can classify two subecotopes of MRP ecotope with traditional RPPs (Figures 3(a) and 3(b)); both subecotopes A1 and A2 depend radically on the infestation or reinfestation of* Anopheles *vectors. The primary vectors include* An. dirus*,* An. minimus*, and* An. maculatus* [32]. The secondary vectors include* An. aconitus*,* An. pseudowillmori*, and* An. sundaicus* [32]. The suspected vectors included* An. barbirostris*,* An. swadwongporni*,* An. philippinensis*,* An. culicifacies*, and* An. campestris* [32]. To appreciate how the probable human-vector contact occurs, we need to comprehend the biology and ecology of local* Anopheles* vectors adapted to local environments.

**Figure 4:**
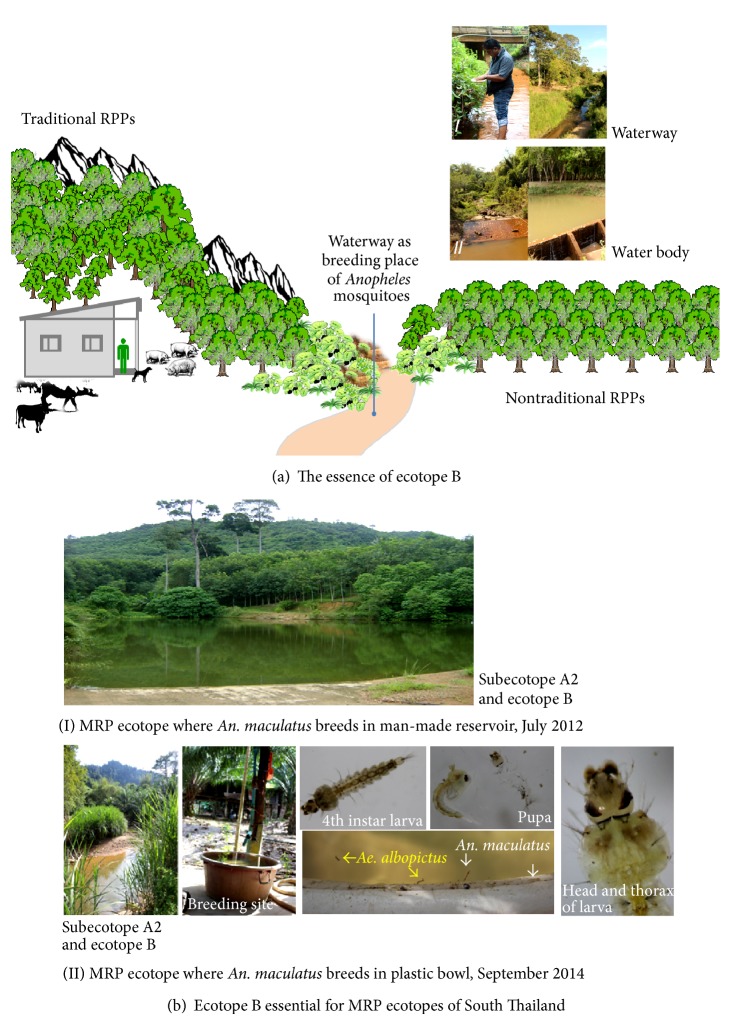
The ecotope B. (a) The essence of the ecotope B. In a given MRP ecotope with traditional or nontraditional RPPs, the water body or the waterway can serve as breeding site for* Anopheles* mosquitoes including potent vectors although adapted to local environments. Most waterways (I) and water bodies (II) are considered the sentinel sites used in the* Anopheles *larval survey. (b) The ecotope B as essential for the MRP ecotope with traditional RPPs in southern Thailand. Evidently, a 2012–2014 ground survey including* Anopheles* larval survey demonstrated that the man-made reservoir (I) as well as the plastic bowl (II) serves as breeding site for* An. maculatus*.

The subecotope A1 (Figure 3(a)) is the MRP ecotope with traditional RPPs that has an extremely high potential for the establishment of MIP—whereby the persistence of breeding sites and feeding activities of* Anopheles* vectors exit in rubber plantation polygons. Rubber plantations—whether mixed with oil palms, fruit orchards, or other perennial trees—can promote the infestation or reinfestation of* Anopheles *vectors. The subecotope A1—in which land use types are heterogeneous—is located at less than 200 MASL or between 100 and 200 MASL, around or near the forests. Similar to that of the subecotope A1, the subecotope A2 has a high potential for the establishment of MIP and is located at less than 100 MASL. The presentation of the subecotope A2 is shown for Phang-nga (Figure 3(a)) and Trat (Figure 3(b)). Also, the existence of the ecotope B, namely, waterway or water body, plays the significant role for the infestation or reinfestation of* Anopheles *vectors (Figure 4). Waterway is a narrow pathway of water that is constantly moving, as the current is regulated by season variation or by man-made dam or irrigated water body. Water body is a source of usually fresh water that is issuing, whether naturally or man-made, from the ground, and is an inland body of standing water—whether small or large, or shallow or deep. As essential for the suitable microclimate including humidity and temperature, both waterways and water bodies are required for the adaptation and survival of* Anopheles* vectors. Most MRP ecotopes, which are confined to the TCAs of malaria-endemic provinces of the South and East of Thailand, cover both subecotopes A1/A2 and ecotope B. These ecotopes serve as the places that are connected to establish the MIP(s). Nonetheless, if these ecotopes are more diverse, the probable human-vector contact will more likely be dynamic and, eventually, the MIP will not truly be specific to a rubber plantation polygon.

More specifically, we need to comprehend MRP multifactors such as agrienvironmental climatic conditions and vector population dynamic that are associated with malaria transmission dynamics in the MRP ecotope with traditional RPPs. In most land areas of traditional RPPs in the South (Figure 3(a)) and East (Figure 3(b)), the normal growth of clonal rubber trees requires agrienvironmental climatic conditions such as heavy rain (average annual precipitation: >1,400 mm), optimal temperature (average annual temperature: approximately 26–28°C), and optimal humidity (average annual humidity: >65%). Only the rain is essential for rubber trees to normally grow. Meanwhile, if there exist the reservoir and irrigation surrounding or within the MRP ecotope (Figure 4(a): Panel II), the agrienvironmental climatic conditions will promote the infestation or reinfestation of anopheline mosquitoes including malaria vectors.

As for malaria vector surveillance in the MRP ecotopes with traditional RPPs, we also leverage needed data/information of what are relevant to the infestation of primary malaria vectors and what are epidemiologically linked with malaria transmission risks. Still, diverse groups of* Anopheles* vectors can be adapted to local environments favorable to breeding and foraging in both subecotopes A1 and A2. For example, the ground surveys in 2014 demonstrated that most MRP ecotopes with traditional RPPs are infested with the* Anopheles *vectors. The suspected rather than primary/secondary vectors are predominantly found in both subecotopes A1 and A2 as shown in Figures 3(a) and 3(b). The abundance and distribution of three primary vectors, including* An. minimus*,* An. maculatus*, and* An. dirus*, seemed to be regulated by seasonal and geospatial variations as similar to that observed by several reports [34–37].

As shown in Figures 1 to 3, most MRP ecotopes with traditional RPPs are close to forests or confined to the valleys or sloping hills that exit waterways at the altitude less than 100 MASL or between contour lines, 100 to 200 MASL. There exist a variety of adult numbers and densities of* An. barbirostris *and its counterparts such as* An. minimus*,* An. maculatus*,* An. dirus*, and* An. aconitus*, all of which are commonly found—through both indoor and outdoor collections using human landing catch—in a wide range of densities between dry and wet-cool seasons. If confined to the valleys or sloping hills at the altitude less than 100 MASL, there exist as many as adult numbers and densities of* An. barbirostris* in some MRP ecotopes, but varying numbers and densities of* An. minimus*,* An. maculatus*, and* An. aconitus* and to lesser extent of *An. dirus* that may disappear or appear intermittently.

More interestingly,* An. stephensi* can breed in widespread breeding places both in the urban and rural settings in South and West Asia [38, 39]. In the rural settings, the larvae can be found in stream pools and margins, ponds, puddles, irrigation channels, seepage canals, catch basins, and springs. In the urban climates, they can be found in a variety of artificial containers such as cisterns, wells, tubs, ornamental ponds, fountains, and sewage. By contrast, a variety of* Anopheles *vectors—which are endogenous to MRP ecotopes with traditional RPPs in the South and East of Thailand—prefer breeding in natural breeding habitats in the wild rather than breeding in artificial containers in the wild or rural and urban settings.* An. minimus* and its counterparts such as* An. maculatus*,* An. aconitus*, and* An. barbirostris*, but not* An. dirus*, are likely to breed their progeny in the waterways such as brook and/or stream. The abundance and distribution of* Anopheles* larva populations found in natural breeding habitats are dynamic due to seasonal and geospatial variations. Among these vectors,* An. maculatus* is a common vector that distributes widely across the South of Thailand and Peninsular Malaysia. But changes in its breeding characteristics are still studied. In Malaysia,* An. maculatus* can breed in the wild in various breeding habitats such as water pockets formed on the bank of rivers and waterfalls, shallow pools, and slow flowing streams—generally located at 100–400 m from the nearest human settlement [40]. Based on* Anopheles* larval surveys, if adapted to local environments that similarly happened in the wild,* An. maculatus* can breed in the man-made reservoir (Figure 4(b): Panel I) and artificial container like plastic bowl (Figure 4(b): Panel II).* An. maculatus*—which is endogenous to the subecotope A2 of Bang Ma MRP ecotope (Figure 3(a))—is adapted well to breed its progeny in a water-containing plastic bowl, as well as to cobreed with* Aedes albopictus*. It is very interesting to note the colonization of two taxa in this niche, showing that* An. maculatus* progeny which has one 3rd larva and six 4th instar larvae accompanies with more than 50 larvae of* Ae. albopictus*. Not only does* An. maculatus* cohabit with* Ae. albopictus* in plastic bowl but also its changes in breeding characteristics remain to be established.

### 4.2. MRP Ecotopes with Nontraditional Rubber Plantation Practices

The MRP ecotopes with nontraditional RPPs have been resulted from the agricultural intensification of rubber plantations instead of crop plantations and other perennial agriculture in the Northeast, North, and Central Thailand, as shown in Figures 1(c) and 3(c). Many rice fields, mixed orchards, or crop plantations in upland areas of the Northeast, North, and Central Thailand have been converted to the newly planted areas of rubber plantations. Still, nontraditional RPPs require suitable agrienvironmental climatic conditions. For instance, it requires average annual precipitation, approximately 1,200–1,400 mm, but annual average for total rainfalls, approximately 120–150 days. Unlike in traditional RPPs, the seasonal harvestation of the natural rubber in the nontraditional RPPs normally starts from March to October (or 8 months of harvestation) while recovering from November to February of the next year (or 4 months of recovery). As with land and water management strategies, the MRP ecotopes with nontraditional RPPs are likely to interconnect human settlements and activities and vector biology and ecology.

The nontraditional RPPs are likely to plant the rubber trees especially confined to riverine or irrigated areas such that the suitable microclimate will eventually create the rubber plantations with dense canopy or shaded environment. Subsequently, the MRP ecotope with nontraditional RPPs nurtured with waterway and/or water body is linked with the adaptation and diversification of anopheline taxa including malaria vectors that maybe develop gradually the species richness (the number) and evenness (the abundance). If microenvironments are managed to resuscitate this MRP ecotope with nontraditional RPPs, a plethora of* Anopheles* mosquitoes will be proxy measure of the infestation or reinfestation of* Anopheles* vectors. Eventually, the reemergence of introduced malaria that possibly occurs in the MRP ecotopes with nontraditional RPPs is explained by the interconnections underlying the dynamics of malaria transmission, the geographical distribution of* Anopheles* vectors, and the susceptibility of human populations.

The baseline entomological data are needed for malaria vector surveillance and control to determine the extent to which the infestation and reinfestation of* Anopheles* vectors occur in newly planted areas before and during the seasonal harvestation of the natural rubber. Continuations of larval survey along with environmental observations will help explain temporal and spatial distribution of local* Anopheles *mosquitoes that can infest in waterways or water bodies confined to newly planted areas as in Figure 4(a) (Panels I and II). The waterway rather than water body serves as breeding site for* Anopheles* vectors is commonly found to be slow-running brook or stream with the vegetation. Nonetheless, it is very difficult to estimate the retention time of egg hatchability and development of first to fourth instar larvae due likely to the species diversity and season variation. However, both larva and pupa stages of* Anopheles *spp. are likely to distribute along the brook or stream margins with the vegetation during wet-cool season, as well as to aggregate in small pools or water pockets close to the margins of the brook or stream during dry season. Particularly in dry season during which the stream normally runs very slowly,* Anopheles *larvae can be found in shallow sand or mud beds of the brook or stream whether or not the aquatic plants or plant debris are present. During wet-cool season, the infestation of* Anopheles* spp. can exhibit diverse groups of* Anopheles* spp. including both nonpotent and potent malaria vectors. In general, seasonal regulation of local* Anopheles *mosquitoes adapted to local environment is an ecological driver that influences the abundance, distribution, and survival of* Anopheles* larvae.

Regarding this, we can therefore classify two subecotopes of the MRP ecotope with nontraditional RPPs (Figure 3(c)); both subecotopes C1 and C2 depend radically on the infestation or reinfestation of* Anopheles *spp. that are adapted to local environments.

The subecotope C1 is the MRP ecotope with nontraditional RPPs that has a moderate-to-high potential for the establishment of MIP—whereby the availability of breeding sites and feeding activities of* Anopheles* vectors exists in rubber plantation polygons. Rubber plantations—whether or not mixed with fruit orchards, forested plantations, and other field crops—can manipulate* Anopheles* infestation or reinfestation. As seen in Figure 3(c), most rubber plantation polygons are located at less than 200 MASL, around or near the forests although nurtured with the waterways. In most TCAs in the Northeast, North, and Central Thailand, many MRP ecotopes with nontraditional RPPs are around or close to the forests to which* Anopheles *vectors are sessile. Also, the malaria incidence is epidemiologically linked with currently developed malaria cases or history of malaria infections in the past.

Based on our experiences, malaria vector surveillance in some sampled sites of the subecotope C1 of Kanchanaburi (Figure 3(c)) showed that both nonpotent and potent* Anopheles *spp. can infest newly planted areas of MRP ecotopes with nontraditional RPPs. There is the likelihood that the subecotope C1 corresponds to the abundance and distribution of diverse* Anopheles* taxa including primary vectors,* An. dirus* and* An. minimus*, and suspected vector like* An. barbirostris*. Larva numbers of* Anopheles* spp. can be found both in dry and wet-cool seasons in waterways and/or water bodies as in Figure 4(a). Among the adapted taxa, larva numbers of* An. barbirostris* and* An. minimus* are more likely to be found in slow-running streams in dry season than in wet-cool season. Perhaps water flows and diets influence the abundance and distribution of larvae of these two species. Conversely, there exist adult numbers and densities of* An. barbirostris*,* An. minimus*, and* An. dirus* whether or not the indoors or outdoors collections by human landing catch are performed during wet-cool season.

The subecotope C2 is the MRP ecotope with nontraditional RPPs that has a low potential of the establishment of MIP—whereby the availability of breeding sites and feeding activities of* Anopheles* vectors exists irregularly in rubber plantation polygons. Rubber plantations—whether or not mixed with fruit orchards, rice fields, and other field crops—can manipulate the* Anopheles* infestation or reinfestation. This ecotope—of which any autochthonous malaria cases are absent—is located at less than 100 MASL on upland crop plantation areas with the irrigation as in Figure 4(a) or confined to the riverine or irrigated areas with agricultural practices. The subecotope C2 can reflect the land use change in the newly planted areas confined to the Northeast, North, and Central Thailand which are unknown or nonendemic for malaria. For instance, many MRP ecotopes with nontraditional RPPs in the Northeast are close to Thai-Cambodia border or Thai-Lao border or Mekong River. Based on our malaria vector surveillance data, both* Anopheles* larvae and adults can be found in some sampled sites of the subecotope C2. The suspected potent malaria vectors include* An. barbirostris* and* An. philippinensis*. Diverse groups of refractory or nonpotent species include* An. jamesii*,* An. hyreanus*,* An. hyrcanus*,* An. subpictus*,* An. sinensis*,* An. nigerrimus*,* An. nivipes*,* An. vagus*, and* An. splendidus*. Among these taxa, adults of* An. barbirostris* and its counterparts such as* An. jamesii*,* An. hyreanus*, and* An. hyrcanus *can be commonly found during the night time than during the early sunset or during the early morning. Both outdoor and indoor collections of* Anopheles* adult mosquitoes can be performed on this nocturnal appearance during which continuations of adult survey along with environmental observations are conducted. The abundance and distribution of* Anopheles* adult mosquitoes obtained by adult vector survey are likely to correspond to that entomological data obtained by larval survey in this subecotope.

## 5. Profiling of MRP Ecotope and Malaria Infection Pocket

### 5.1. Identification and Characterization

As to bridge malaria landscape ecology and epidemiology [41, 42], the MRP ecotope can be defined as a fundamental land unit, or it is considered as small as landscape scale of analysis used in landscape ecology [43–45]. The MRP ecotope (spatial) is suited to determine the degree to which the agricultural land use changes attributed to the expansion of rubber plantations (nonspatial) have the potential effects on malaria transmission risks. The MRP ecotoping, on the other hand, can also serve the holistic assessment framework whether such negatively ecosystemic outcomes contribute substantially to malaria transmission dynamics in Thailand and, internationally, within the other GMS countries. As mentioned earlier, the relative land use types of the MRP ecotope including rubber plantation polygons, waterways, and water bodies serve as the spatial data set—which are essential for the identification and characterization of MRP ecotope confined to the TCA, based on both land use and contour maps. The identification is to define a land area geographically associated with the infestation or reinfestation of* Anopheles* vectors. The characterization is to define a land area that has the potential risk for malaria transmission.

**Figure 5:**
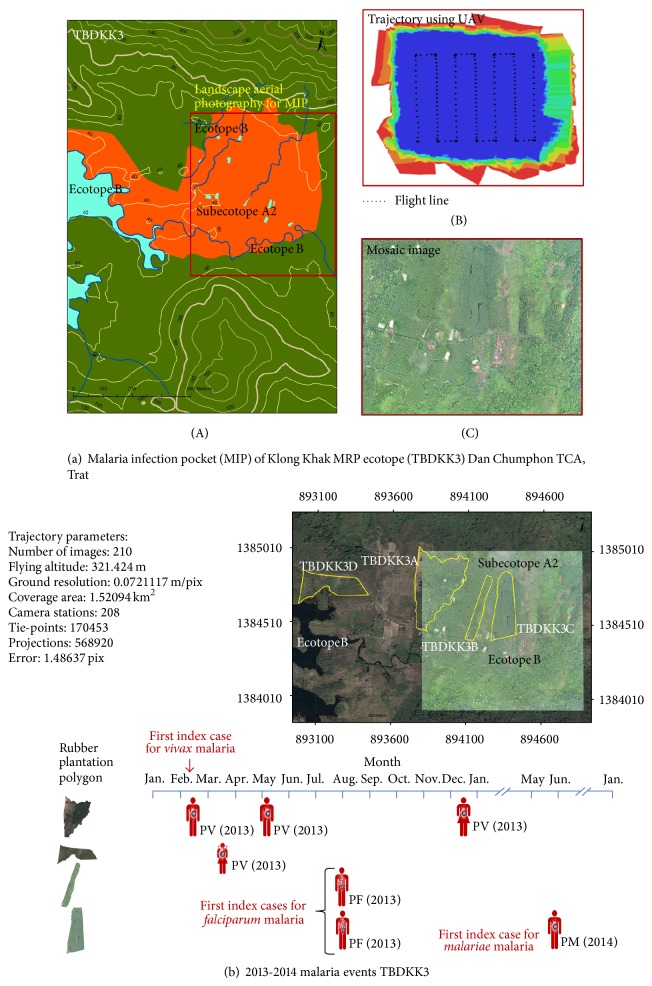
Profiling of MRP ecotope and MIP. (a) A profile of Klong Khak MRP ecotope confined to the Dan Chumphon TCA, Trat province, can be step-by-step processed: (A) identification and characterization of GIS-based MRP ecotope using both topographic land use and contour maps; (B) landscape aerial photography that employs planned trajectory of georeferenced MRP ecotope using the multirotors as the UAV; and (C) construction of image mosaics using a feature-based technique for the joint estimation of all snapshots. (b) A profile of the MIPs confined to the Klong Khak MRP ecotope can be spatially perspective to determine the degree to which the probable human-vector contact is associated with malaria infection. All the validated land use maps illustrated with contour lines were constructed as before. Moreover, topographic presentations of the spatial data shown in (a) and (b) can be manipulated using the ArcGIS applications to monitor the infestation or reinfestation of* Anopheles* vectors as well as the malaria event in a time-series manner.

For instance, the topographic land use maps employ levels I to III land use information originally obtained from the satellite imagery. When analyzed for any TCA at the subdistrict or village level, the landscape features of the MRP ecotope exhibit the degree to which* Anopheles* vectors can infest or reinfest in respective valleys or sloping hills covered with rubber plantations and nurtured with waterways and/or water bodies. Basically, the malaria control stratification system used in the registry of the TCA at the subdistrict or village level should be currently or up-to-date reviewed by the NMCP's implementers or the infection control personnel as seen in Figure 1. Based on the landscape parameters or spatial and nonspatial data available for the delineation of the MRP ecotope, a contour map of the TCA illustrated with contour lines will better help us to permit the coverage land area infested with* Anopheles *vectors as seen in Figure 3, in the presence or absence of any currently developed malaria cases. As seen in Figures 2(b), 3, and 5, the landscape structure of* Anopheles*-infested land area is relatively related to the specific ridge of hills with low-to-moderate steepness of the slope that generate waterways, especially brooks and streams, or perhaps fork. More essentially, hillside slope and valley(s) confine the sloping waterways that move slowly down or flowing downward on a gap or pass. If covered with rubber plantations mixed or not mixed with other perennial trees and orchards, the responsible valleys or hills can confine the MIP of corresponding rubber plantation polygon. However, the ground truths—especially for which the attribute data of probable human-vector contact are relevant to rubber plantations polygons—are needed to demarcate the georeferences using the GPS and to triangulate the spatial data obtained from the standard land cover classification systems of remotely sensed satellite imagery [17, 46] or landscape aerial photography.

### 5.2. Landscape Aerial Photography

The landscape aerial photography (LAP) is the advanced technology of aerial imagery of the perspective landscape scale such that the UAV can provide high resolution of land use map as compared to that created by the remotely sensed satellite imagery (Table 1). The LAP—which is applied to a coverage area map of this georeferenced MRP ecotope that established the malaria events between 2013 and 2014 (Table 2)—is demonstrated by using UAV and followed by the programmed trajectory [47–49]. The UAV using the multirotors employs the projective camera that virtually provides a coverage area map pertaining to a series of aerial photos with high resolution. More significantly, the spatial data can be deduced from any locations at which the ground survey cannot reach. As seen in Figures 5(a) and 5(b), the multirotors programmed with the trajectory can perform aerial imagery survey in which the on-board camera can record a series of 210 snapshots over a coverage area of 1.5 km^2^ at the altitude above 300 meters. The Klong Khak MRP ecotope encompasses the unique landscape features of the expansion of the rubber plantations on the valleys and hills infested with* Anopheles* vectors (Figure 5(a)) and has the high potential for malaria transmission (Figure 5(b)) that exhibits the MIPs within a 2 km^2^ land area.

### 5.3. Image Processing

Mosaic techniques are essential for UAV image processing and construction of a mosaic image to obtain a broad landscape, based radically on the georeference and anchor image of the partially aerial snapshots (Figure 5). Because there are pairs of partially uncalibrated snapshots that overlap weakly perspective views, image mosaics of corresponding sidelap and overlap views are analyzed to present pairs of strongly perspective views relevant to the georeference. A feature-based technique [50–52] is commonly referred to as a linear feature-based noniterative method for the joint estimation of all images. The technique is the most widely used to perform image registration, that is, pairwise registration of the anchor images and between pairs of the nonanchor images, and hence construction of registered image pairs used in the joint algorithm. As seen in Figures 5(a) and 5(b), constructing image mosaics from 210 snapshots is an essential step for leveraging spatial data whether or not the MRP ecotope has the unique landscape features pertaining to the establishment of MIP.

### 5.4. Visualization and Interpretation

Based on the construction of image mosaics mentioned above, a high-resolution perspective view represents a present landscape structure of the MRP ecotope or MIP confined within the MRP ecotope. The ground surveys are also needed to validate the spatial data obtained from LAP using UAV. The UAV-based maps are georeferenced using the GPS; that is, both validated spatial and nonspatial data can be manipulated precisely and timely as described below.

More evidently, Table 2 and Figure 5(b) show a profile of the Klong Khak MRP ecotope that exhibits the 2013-2014 events of the malaria infections in relation to rubber plantation practices. The infographic presentations of the MIP confined to this Klong Khak MRP ecotope can be performed on the different platforms of the GIS-based UAV and Google Earth applications. As in Figure 5(b), the validated spatial data initially obtained by the UAV-based LAP and mosaic technique are displayed on the ArcGIS software applications or the Google Earth applications and then synchronized with the attributes of malaria events. Regarded as the relative land use type of the Klong Khak MRP ecotope, the rubber plantation polygons correspond to probable human-vector contact sites because routine RPPs during the night time by rubber farmers or rubber plantation workers rendered them susceptible to multiple bites of* Anopheles* vectors including infective bite(s) at multiple locations. Obviously, different rubber plantation polygons contribute to epidemiologic patterns of the infection over a time period. Malaria transmission occurs as the event of* Plasmodium* infection, whether single or mixed, in a point manner or an intermittent manner rather than the sequential event of a continuous infection. Malaria infection is considered as an individual newly infected with either of four* Plasmodium* spp. based on blood examinations. This MRP ecotope exhibits the strong evidence that the availability of breeding sites and feeding activities of primary* Anopheles* vectors relates to the single malaria infections, that is, any individuals infected with the only one type of the* Plasmodium* species over a time period. That is, this MRP ecotope relates the occupational and behavioral exposures to render the adults susceptible to outdoor bites of* Anopheles* vectors including infective bite(s). The childhood infection may be acquired through outdoor biting if the children or younger persons are accompanied persons involved in RPPs during the night time.

Like the forest-related malaria in Thailand, the MRP ecotope exhibits seasonal malaria transmission; that is, the incidence is relatively higher during wet season than during dry season and hence it increases with increasing age. Once seasonal transmission of autochthonous malaria occurs in the MRP ecotopes that are confined to the TCA at the subdistrict level, it does not mean that all the malaria infections are epidemiologically linked with the same source of the infection. Thus, in a given MRP ecotope, malaria transmission occurs as the event of the infection in one or more MIPs. Moreover, the profiling of the MRP ecotope and MIP could benefit the advantages of UAV imagery rather than the remotely sensed satellite imagery (Table 1). Still, the systemic and uniform approaches to MRP ecotoping require both the epidemiological data obtained from routine malaria surveillance systems and household surveys and the entomological data obtained by* Anopheles* larva and/or adult surveys.

## 6. Perspectives

The rubber forestry is a land management strategy by which the people benefit from the exploitations of propagating rubber plantations and harvesting the natural rubber and wood. Thailand is a case study such that there are coincided phenomena that land use/land cover changes influenced by increasing land areas for the rubber forestry are related to increased risks for malaria although the vertical implementation of the NMCP has been gradually achieving the overall reduction of malaria mortality and morbidity, and the program is moving toward the preelimination phase of malaria control. The NMCP employs the malaria control strategies suited to stratify the TCA at the subdistrict or village level. This stratification is based radically on both the epidemiological data obtained by the malaria surveillance systems that employ the notification of laboratory-confirmed malaria infections or case numbers reported by active and passive surveillance systems and the entomological data obtained by the malaria vector surveillance that monitors the infestation/reinfestation of* Anopheles* vectors. However, malaria transmission dynamics occurs continually in the TCAs where the MRPs coexist with the changes of malaria landscape ecology and epidemiology. More importantly, the effective and sustained primary prevention strategies are still desired because the people involved in the rubber forestry sector do not know what renders them susceptible to the malaria infections. Regarding this, if large-scale malaria control is required, the landscape of forest-related or forest fringe-related malaria needs to be logically analyzed in completeness, correctness, and timeliness.

### 6.1. Ecotope-Based Entomological Surveillance

As for malaria vector surveillance that achieves the targets or desired outcomes, the ecotope-based entomological surveillance (EES) can provide the proof that the MIP is the hotspot suited to determine the extent to which the responsible* Anopheles *vectors and their counterparts can infest or reinfest within the MRP ecotope as described elsewhere [32]. From global and regional perspectives, the GMS countries are being shifted to the world largest natural rubber producers that exhibit growing trend of increase in land areas of rubber plantations [27], as the coordinators of the NMCPs and other public health personnel involved in policy formulation and strategic deployment might have adopted the interventions and services suited to the target populations in the rubber forestry sector. As mentioned earlier, such EES approaches can provide the strong evidence that the informative malaria ecotope can be modeled for malaria transmission dynamics to link between human-vector-parasite interactions and human-environment-vector interactions in receptive areas of land use and land cover change [32]. Nonetheless, the EES requires the needed data/information of the malaria infections or the incidence in order to analyze the vulnerability in how the diverse groups of* Anopheles* vectors potentially transmit the malaria parasites vertically to humans in the MRP ecotope, based on the time lag and probable human-vector contact.

### 6.2. Global Platform of MRP Ecotope

The GIS-based MRP ecotoping is the integrated approach suited to confine MIP—by which once the establishment of MIP is monitored within the TCA or across TCAs at the subdistrict level. In addition to the approaches and methods described elsewhere [41, 42, 53], this approach can be optionally applied to or used as a promising tool for the identification and characterization of MIP. If properly validated, this approach will ease decision-making process of prioritizing most risk areas; that is, this leads to the right direction of the selection of management activities and strategies to be implemented to the target areas and populations at risk by the national-level and subnational-level coordinators of the NMCP. Also, it will help develop the downstream protocols, procedures, and tools for decision-making implementation, evaluation, and monitoring of the effectiveness of those selected strategies and services that can lead to the reduction of the operational costs for surveillance, prevention, and control.

The delineation of MRP ecotope can be optionally suited to determine the scope of very large infographic data relating to human, agent, vector, and environment. In practice, the scalability of MRP ecotope can be georeferenced by using the grid assignment. For example, a 1 × 1 km grid of the MRP ecotope that corresponds to the latitude and longitude can be used as small as landscape scale or the unit of assessment of the malaria infections in humans and* Anopheles* vectors as well as of insecticide resistance in* Anopheles* vectors. As shown in Figure 3, the contour maps show the scalable MRP ecotopes cover geographically defined transmission area of as small as 500 m^2^, in which at least one MIP exists. The configuration of MRP ecotope map illustrated with contour lines allows us to infer the parameters specific for* Anopheles* vector-infested rubber plantation polygons that lie between successive contour lines. More interestingly, such specific locations to which rubber plantation polygons on the valleys or sloping hills and the steepness of slopes are confined are likely to regulate the abundance and distribution of potent* Anopheles *vectors between adjacent contour lines. The operational and research endeavors for malaria prevention and control should be paid attention to harmonizing the global platform of the georeferenced MRP ecotope, whether or not it is mapped on different platforms of applicable GIS. In particular, the NMCPs can adopt the georeferenced MRP ecotope through the development of malaria ecotope database and management when the TCA at the subdistrict or village level is logically analyzed and targeted for the preelimination and elimination phases.

## Supplementary Material

Table S1 provides the current status of land use information among five regions of Thailand, 2009–2012.

## Figures and Tables

**Table 1:** The disadvantages and advantages of remotely sensed satellite imagery and UAV imagery.

Criteria	Satellite imagery	UAV imagery
(1) Scalability	Largely perspective landscape scale	Smaller perspective landscape scale; the UAV whose flying altitude is at 300 meters of scales to a coverage area of 1.5 to 2 km^2^ per fight for the multirotors or up to 5 km^2^ per fight for the fixed wing

(2) Coloring and brightness	Equitable image as often the cloud cover does not permit virtually clear vision	No equitable image but less likely to be deviated by the cloud cover; a series of aerial photos taken by the projective camera requires time-consuming

(3) Precision	Highly accurate and georeferenced image	Based on the calibration of georeferenced image before UAV image processing

(4) Ground resolution	Highly definite but very difficult to adjust the resolution	Easily adjusted to the desired resolution based on flying altitude

(5) Cost	Too costly as the purchase order usually requires time-consuming	Low cost and less time-consuming

(6) Timeliness	Outdated	Timely as desired

**Table 2:** A profile of the Klong Khak MRP ecotope^a^ that exhibits malaria infections in relation to rubber plantation practices during a two-year period, 2013-2014.

Patient ID	Age (yr)	Gender	Type of infection	Day of illness	Day of diagnosis	Time lag (month)
TBDKK3A1^b^	33	M	PV	16 February 2013	18 February 2013	0
TBDKK3D1	6	F	PV	17 March 2013	19 March 2013	1
TBDKK3A1^b^	33	M	PV	7 May 2013	7 May 2013	3
TBDKK3B1	25	M	PF	30 July 2013	1 August 2013	0
TBDKK3C1	40	M	PF	30 July 2013	1 August 2013	0
TBDKK3A2^c^	52	F	PV	17 December 2013	17 December 2013	7
TBDKK3C2	27	M	PM	28 May 2014	29 May 2014	0

^a^MRP ecotope code—TBDKK3.

M: male, F: female, PF: *P. falciparum*, PM: *P. malariae*, and PV: *P. vivax*.

^b^First index case for the MIP who developed relapse of *P. vivax* within 3 months after radical first-line treatment using 2,500 mg chloroquine and 210 mg primaquine.

The initial time lag, 0 months, refers to the day of diagnosis for any type of the *Plasmodium* infection for ^b^the first index case up to that of ^c^any sequential case with the same type of the *Plasmodium* infection within a year.
