# Supplementary material for: Landscape Ecology and Epidemiology of Malaria Associated with Rubber Plantations in Thailand: Integrated Approaches to Malaria Ecotoping
Source: Interdiscip Perspect Infect Dis. 2015 Mar 9;2015:909106. doi: 10.1155/2015/909106 (PMC4370114; doi:10.1155/2015/909106)
Supplement: Supplementary file 1 — Table S1 provides the current status of land use information among five regions of Thailand, 2009–2012. [file 909106.f1.pdf]

Table S1 Current status of land use* among five regions of Thailand, 2009-2012

Land use type	North	Northeast	Central	East	South
**Agricultural land**	6,368.6 (37.5)	11,415.1 (67.6)	3,425.6 (49.3)	2,148.5 (62.5)	4,192.9 (59.3)
Paddy field	2,626.8	7,340.8	1,424.1	487.7	381.9
Field crop	2,034.6	2,602.1	1,078.1	556.0	2.9
Perennial[a]	400.5	1,164.5	264.1	638.6	3.310.7
Orchard	569.2	188.2	357.4	319.7	404.5
Horticulture	37.3	23.6	72.6	4.1	3.9
Swidden cultivation	652.7	0	0	0	0
Pasture and farm house	32.2	71.5	29.1	15.4	1.9
Aquatic plant	0.1	0.1	2.0	0.1	0.1
Aquaculture land	12.0	20.7	198.2	126.9	86.8
Integrated farm/ Diversified farm	3.2	3.6	0	0	0.2
**Forest land**	9,266.8 (54.6)	3,148.5 (18.7)	2,385.5 (34.3)	788.0 (22.9)	2,062.7 (29.2)
Perennial forest	617.3	2,272.5	404.0	744.6	198.8
Disturbed forest	8,649.5	876.0	1,981.5	43.4	1,863.9
**Urban and built-up land**	602.6 (3.6)	868.3 (5.1)	617.2 (8.9)	235.3 (6.8)	265.6 (3.7)
**Water bodies**	228.9 (1.4)	562.6 (3.3)	232.5 (3.3)	84.5 (2.5)	296.2 (4.2)
**Miscellaneous land**	497.5 (2.9)	890.9 (5.3)	291.3 (4.2)	181.8 (5.3)	254.2 (3.6)
Others	451.6	752.7	254.1	160.4	203.4
Marsh and swamp	45.9	138.2	37.2	21.4	50.8
**Total**	16,964.4	16,885.4	6,952.1	3,438.1	7,071.6

*Land area in hectares (ha x 1000) and percent of land area in relation to land use type in parentheses are shown. With respect to land cover classification system (LLCS) available at http://www.fao.org, the levels I to III land use information were adapted from the Land Development Department, Ministry of Agriculture and Cooperatives available at http://www.ldd.go.th/.

[a]Perennial agriculture including mixed perennial grains/oil seeds/legumes/forages/trees can exploit the benefits from both upland and lowland intensification of rubber tree, oil palm, eucalyptus, teak, coffee, tea, mulberry, bamboo, kapok, betal palm, rain tree, and so on.
